# Immune checkpoint inhibitor therapy for advanced HPV-related penile squamous cell carcinoma: a rare case report

**DOI:** 10.3389/fonc.2025.1569124

**Published:** 2025-07-03

**Authors:** Zhen-Kun Pan, Meng-Hua Wu, Hua Shi, Jin-Sheng Ye

**Affiliations:** ^1^ Department of General Surgery, Beijing Yanqing Hospital of Traditional Chinese Medicine, Beijing, China; ^2^ Department of Urology, Beijing Hospital of Traditional Chinese Medicine, Capital Medical University, Beijing, China; ^3^ Department of General Surgery/Oncology, Beijing Hospital of Traditional Chinese Medicine, Capital Medical University, Beijing, China

**Keywords:** penile squamous cell carcinoma, human papillomavirus type 16, immunotherapy, lymph node dissection, checkpoint inhibitors, advanced cancer

## Abstract

**Background:**

Human papillomavirus type 16 (HPV-16)-associated penile squamous cell carcinoma (PSCC) poses considerable therapeutic challenges, especially in its advanced stages. Although surgery continues to be the cornerstone of treatment, immunotherapeutic approaches hold a promising alternative for patients unable to endure conventional chemotherapy.

**Case summary:**

A 69-year-old male presented with progressive ulceration of the foreskin over the course of one year, which ultimately extended to the glans, accompanied by inguinal lymph node metastasis. The patient underwent surgical resection, including bilateral inguinal lymph node dissection. Histopathological examination confirmed a diagnosis of HPV-16-related PSCC with concomitant PD-L1 expression. Given the patient’s poor tolerance to chemotherapy, he was treated with four cycles of the PD-1 inhibitor tislelizumab, resulting in a partial response.

**Conclusion:**

This case underscores the promising potential of immunotherapy as a viable alternative treatment for advanced PSCC in patients who are unable to tolerate chemotherapy. The synergistic integration of surgical intervention, immunotherapy, and psychological support is essential to achieving the best possible outcomes for patients.

## Introduction

Penile squamous cell carcinoma (PSCC) is a relatively rare malignancy with a higher incidence in developing countries compared to developed nations, presenting significant therapeutic challenges in clinical management (1). Human papillomavirus (HPV) infection, particularly HPV-16, is the primary risk factor for PSCC, with additional contributing factors including phimosis, recurrent infections, smoking, multiple sexual partners, and low socioeconomic status (2, 3).

Early diagnosis and prompt treatment are crucial for a favorable prognosis. The prognosis for individuals diagnosed with advanced penile cancer, particularly those with distant lymph node metastasis, is typically unfavorable. Therefore, the selection of treatment strategies for these patients is of paramount importance. Despite surgical intervention remaining the primary therapeutic approach, post-operative management of tumor recurrence and subsequent treatment presents significant clinical challenges. Chemotherapy demonstrates limited efficacy, characterized by rapid tumor recurrence and substantial toxicity that critically compromises patient tolerability. Existing literature substantiates that patients with suboptimal chemotherapeutic responses experience markedly reduced overall survival (OS) (4). Concurrently, immunotherapy emerges as a promising alternative, yet its underlying mechanisms and definitive biomarkers remain incompletely elucidated. The precise selection of immune checkpoint inhibitors (ICIs) continues to represent a nuanced clinical dilemma. Notably, while high PD-L1 expression correlates with adverse prognostic indicators, it simultaneously provides compelling evidence supporting potential immunotherapeutic efficacy (5).

This case report presents a 69-year-old patient with HPV-16-associated advanced PSCC who was intolerant to conventional chemotherapeutic regimens. Following PD-1 inhibitor administration, the patient demonstrated partial remission—a clinically active outcome that illuminates the therapeutic potential of immunological interventions in challenging oncological scenarios.

## Case presentation

### Chief complaints

A 69-year-old male patient presented with a one-year history of progressive foreskin ulceration and a six-month history of left inguinal mass.

### History of present illness

The patient first observed localized ulceration on his foreskin approximately one year ago. Despite the application of topical anti-infective treatments, the condition not only persisted but also advanced to involve the glans. On physical examination, an ulcerative, exudative mass was identified in the left inguinal region, accompanied by a malodorous discharge. The patient married at the age of 52 and identifies as heterosexual. He reported experiencing infrequent and less harmonious sexual relations with his wife, compounded by the presence of a phimotic foreskin and suboptimal genital hygiene practices. The patient is in a monogamous relationship and denies engaging in any extramarital sexual contacts.

### History of past illness

The patient, who comes from a low-socioeconomic background, presented with no significant medical comorbidities. He denied any history of chronic conditions such as diabetes, hypertension, etc. that could potentially influence disease progression.

### Personal and family history

The patient’s family history was reviewed, revealing that both parents died of natural causes, with no known history of cancer. He has two healthy sisters who did not report any significant medical issues.

### Physical examination

**Figure 1:**
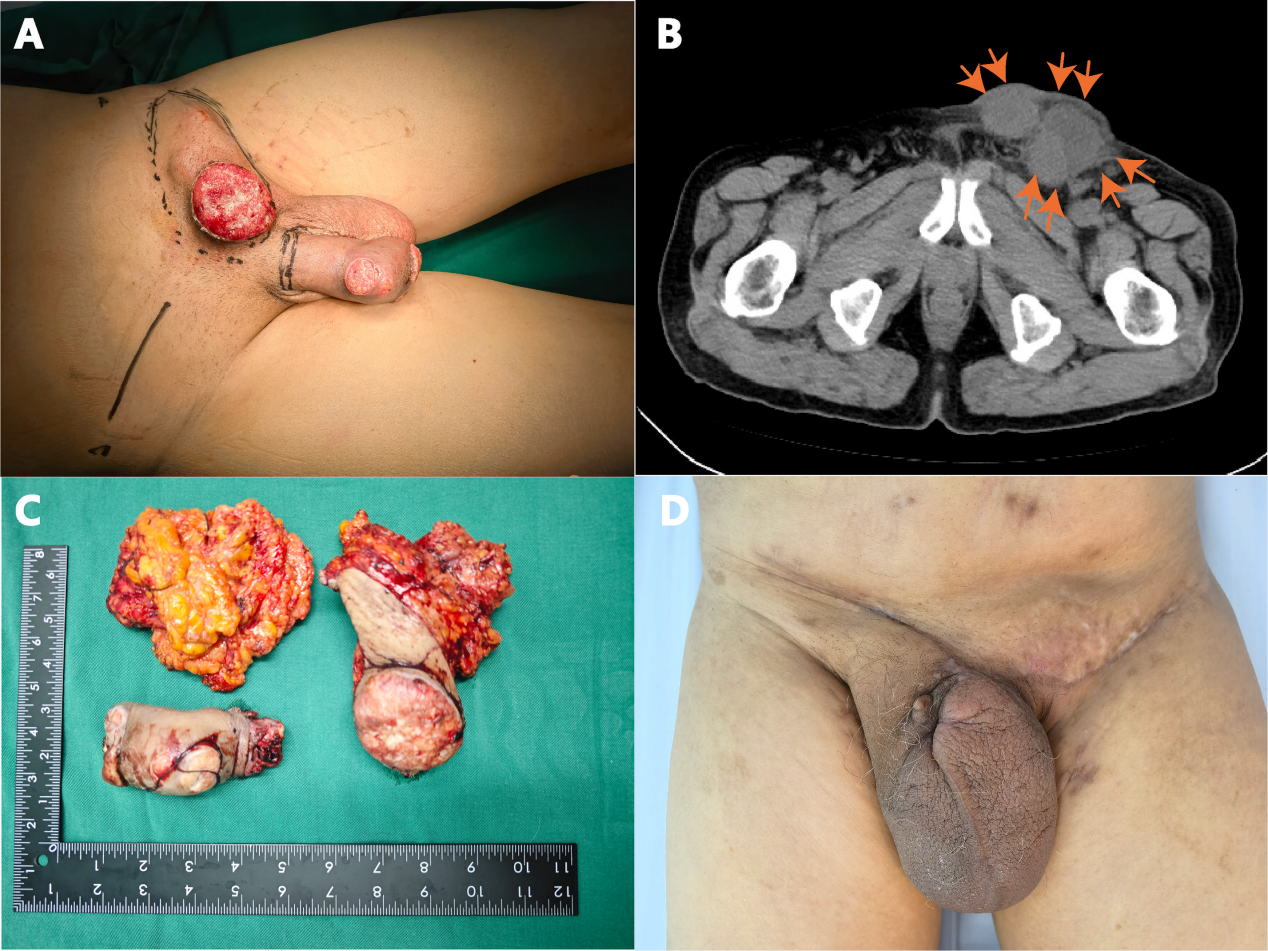
**(A)** Ulcerative lesion on the glans penis and shaft, accompanied by inguinal lymphadenopathy; **(B)** CT scan showing enlarged left inguinal lymph nodes; **(C)** Resected penile tumor and lymph nodes; **(D)** Surgical site healing at 6 weeks post-operation.

Physical examination revealed an elongated prepuce with phimosis, with an ulcerated, exudative mass measuring about 6 × 5 cm on the foreskin. The left inguinal lymph nodes were palpably enlarged about 5 × 4 cm, firm, and fixed to underlying tissue. See (**Figure 1A**). Postoperative pathology results report: Paraffin block number YB00826-24: CD31 (vascular +), CD34 (vascular +), Ki-67 (proliferation index 80%), P16 (diffuse +), P40 (+), P63 (+). The VENTANA PD-L1 (SP263) Assay, produced by Ventana Medical Systems, Inc., is designed for the qualitative detection of PD-L1 protein. However, there are currently no established threshold values for PD-L1 expression in penile cancer in China. Laboratory examinations were within normal ranges as follows **Table 1**.

**Table 1:** Laboratory examinations were within normal ranges.

Category	Result	Normal Range
Hematological Parameters		
White Blood Cell Count	8.07 × 10^9^/L	4-10
Hemoglobin	120 g/L	120-160
Platelet Count	101 × 10^9^/L	100-300
Biochemical Profiles		
Alanine Aminotransferase (ALT)	26.5 U/L	9-50
Aspartate Aminotransferase (AST)	33.6 U/L	15-40
Creatinine (Cre)	61.1 umol/L	57-111
Tumor Markers		
Alpha-fetoprotein (AFP)	10.51 ng/mL	0-12
Carcinoembryonic Antigen (CEA)	2.68 ng/mL	0-5
Cancer Antigen 125 (CA-125)	23.3 U/ml	0-35
Cancer Antigen 153 (CA 153)	6.20 U/ml	0-31.3
Cancer Antigen 199 (CA 199)	12.2 U/ml	0-35

### Imaging examinations

Enhanced CT examination revealed extensive penile involvement with significant left-sided inguinal lymphadenopathy, presenting as a round mass shadow (**Figure 1B**), suggestive of potential malignant neoplasm. The penile tumor was located at the distal end, appearing as a nearly round mass measuring approximately 5.5 × 5.5 cm, while the left inguinal region exhibited a tumor measuring 5 × 4 cm.

**Figure 2:**
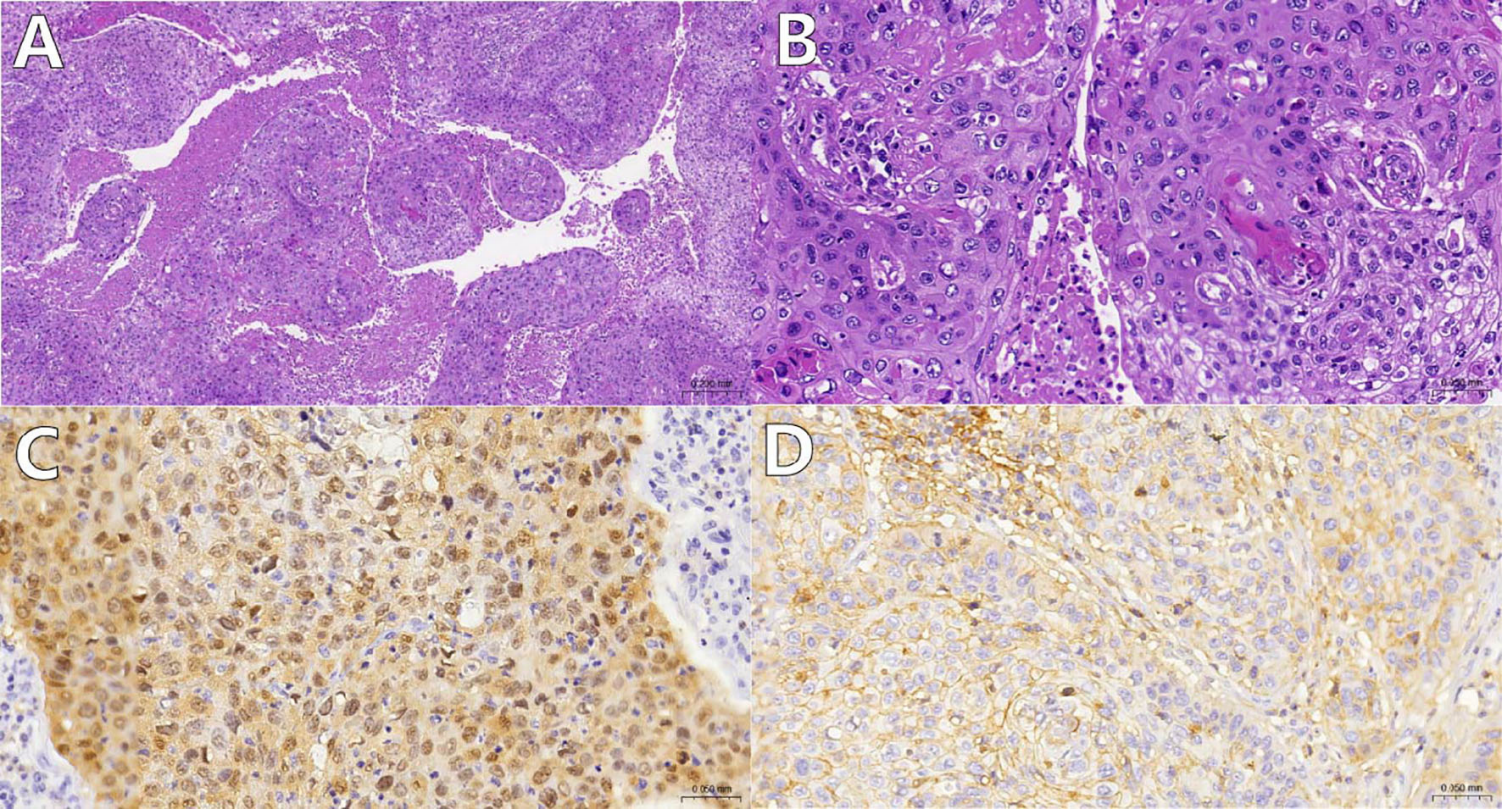
**(A, B)** Histological appearance of tumor cells under low and high magnification using Hematoxylin and Eosin (HE) staining. The tumor tissue grows in a reticular and islet-like pattern, showing extensive necrosis [**(A)**, magnification: 100x]; the tumor cells exhibit significant atypia, with pathological nuclear division and keratinization bead formation visible [**(B)**, magnification: 400x]; **(C)** Tumor cell nuclei and cytoplasm demonstrate diffuse P16 positivity(magnification: 400x); **(D)**:Tumor cells show moderate intensity complete cell membrane staining for PD-L1(SP263)(magnification: 400x).

Pathological examination confirmed HPV-16-related squamous cell carcinoma (**Figures 2A–C**), with positive PD-L1 expression (**Figure 2D**).

## Final diagnosis

HPV-16-related penile squamous cell carcinoma with inguinal lymph node metastasis.

## Treatment

The patient underwent surgical excision of the lesion with bilateral inguinal lymph node dissection. Approximately 1 cm of the penile stump was preserved to maintain normal urinary function. Due to the poor tolerance to the chemotherapy regimen (combination of cisplatin and paclitaxel), the treatment was adjusted to monotherapy with a PD-1 inhibitor. Based on previous literature (6), we selected tislelizumab for four cycles of immunotherapy, achieving a partial response. The patient received tislelizumab at 200 mg intravenously every 3 weeks for four cycles.

## Outcome and follow-up

**Figure 3:**
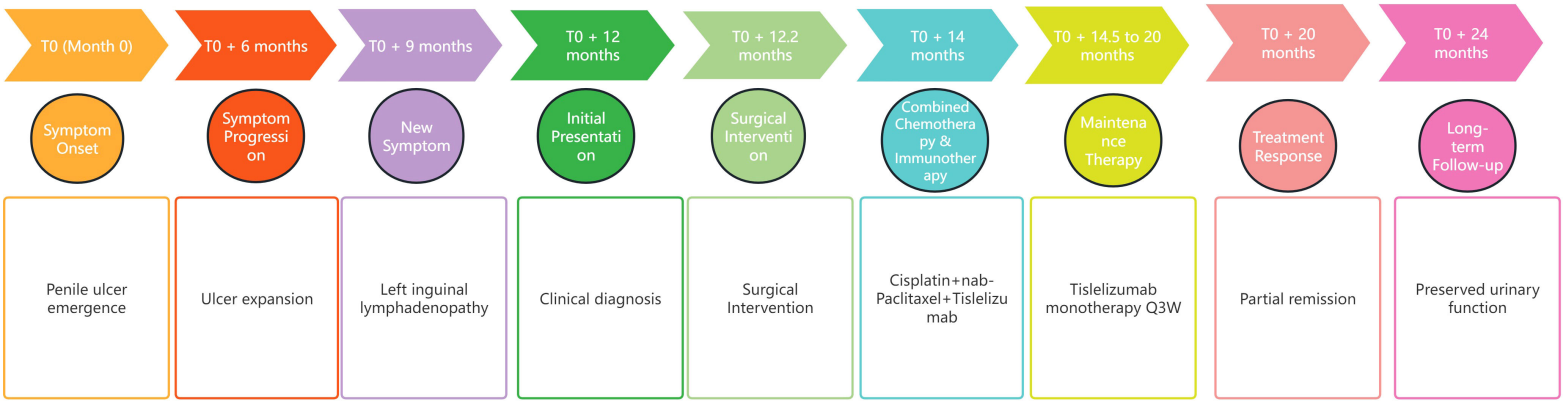
Timeline of the episode of care. This figure organizes the historical and current information from this episode of care into a timeline format, detailing significant events, interventions, and patient outcomes throughout the care process.

The patient exhibited a partial response to immunotherapy. Postoperative complications, such as chylous ascites, were managed conservatively. Following the initial cycle of combined chemotherapy and immunotherapy, the patient experienced severe bone marrow suppression (refer to **Table 2** for detailed hematological parameters). With supportive treatment, bone marrow function gradually improved. During the three subsequent cycles of immunotherapy, no clinically significant bone-marrow suppression occurred; fatigue and poor appetite remained the predominant adverse effects. At the one-year follow-up, his FACT-G (Functional Assessment of Cancer Therapy-General) score was 72, indicating a good overall quality of life. He is able to void spontaneously through the residual urethra without catheter assistance and perform activities of daily living independently, although persistent scrotal edema remains a concern. A timeline summarizing the historical and current information from this episode of care is provided in **Figure 3**.

**Table 2:** Variations in hematological parameters throughout treatment.

Hematological Parameters	Baseline	After 1st Cycle (Chemo + Immunotherapy)	After Supportive Treatment	After 4th Cycle (Immunotherapy Monotherapy)	Normal Range
White Blood Cell Count(× 10^9^/L)	8.07	2.32 ↓	6.67	8.17	4-10
Neutrophils (× 10^9^/L)	4.04	1.14 ↓	5.01	6.51	2-7.7
Hemoglobin (g/L)	120	85 ↓	92 ↓	100 ↓	120-160
Platelet Count (× 10^9^/L)	101	41 ↓	96 ↓	93 ↓	125-350

The symbol “↓” indicates a value below the lower limit of the normal reference range (i.e., decreased).

## Discussion

Approximately 95% of penile cancers originate from squamous epithelial cells, which include both *in situ* and invasive SCC. Studies have indicated that PSCC can be categorized into human papillomavirus (HPV)-related and non-HPV-related types (7). A retrospective study conducted from 1986 to 2008 found that approximately 50% of penile malignancies were HPV-related, primarily HPV-16 (about 60%) followed by HPV-18 (about 13%) (8). Research indicates that patients with HPV-associated p16 positivity typically exhibit more favorable prognoses, characterized by notably superior OS and disease-free survival (DFS) rates in comparison to their HPV-negative counterparts (9). Early manifestations typically comprise localized ulceration or growths on the foreskin or glans. The treatment of penile cancer mainly involves targeting the tumor itself and preserving function, including urinary and sexual functions, which also affects the patient’s psychological state. The physiological organ loss and self-esteem issues post-surgery are significant considerations. At the same time, timely surgical treatment facilitated the radical excision of the lesion and bilateral lymph node dissection, despite the elevated risk of wound healing complications. Prior research has underscored the crucial role of lymph node dissection (10). Comprehensive lymph node dissection can potentially improve patient prognosis and survival. Conserving an approximate 1 cm penile stump ensured normal urinary function and optimized psychological acceptance for the patient (8). Following one cycle of chemotherapy, which could not be sustained due to severe bone marrow suppression, the patient transitioned to single-agent PD-1 immunotherapy, indicating that monotherapy utilizing immunotherapy can be an effective alternative strategy. The use of immunotherapy as a novel and promising treatment approach for advanced PSCC is encouraging (5). Follow-up examinations revealed no discernible metastatic lesions, indicating a partial response. This outcome suggests that single-agent immunotherapy can be an effective alternative when chemotherapy is not tolerated. Research suggests that SCC is highly immunogenic (11). This may be attributed to the distinct properties of the tumor microenvironment (TME), which can effectively stimulate and augment the patient’s immune response against cancer. Future research will aim to optimize genomic stability maintenance to enhance T cell recognition and tumor cell eradication efficiency. The partial remission is likely driven by HPV-16 E6/E7-induced p16INK4a and PD-L1 up-regulation, which enhances antigen presentation and primes tumour cells for PD-1 blockade (12).

Chromosomal instability typical of squamous-cell carcinomas further increases neoantigen load, reinforcing immune recognition, While this biology explains the benefit of tislelizumab, the agent can still provoke early immune-related events-transient myelosuppression, fatigue, and rash have all been recorded within the first month and mirror the mild hematologic suppression and scrotal edema seen in our patient (13). Although HPV-positive PSCC typically exhibits relatively low tumor mutational burden (TMB) and microsatellite instability (MSI), these factors alone do not fully determine the response to immune checkpoint inhibitors. Prior genomic profiling studies indicated that HPV-positive PSCC generally displays lower TMB levels compared to HPV-negative PSCC, where high TMB frequently correlates with increased PD-L1 expression and enhanced immune infiltration (14, 15). Furthermore, MSI-high phenotype is exceedingly rare among PSCC patients, limiting its applicability as a universal biomarker for immune checkpoint blockade (14). The immune microenvironment (TME) also distinctly differs between HPV-positive and HPV-negative PSCC. HPV-negative tumors often demonstrate active immune infiltration with robust PD-L1 expression and higher T-cell activity. In contrast, HPV-positive PSCC typically presents an ‘immune-exhausted’ phenotype characterized by abundant but dysfunctional T-cell infiltration (16). Nevertheless, the immunotherapeutic potential remains promising for HPV-positive PSCC, primarily driven by HPV-associated oncogenic E6 and E7 proteins (16, 17). These viral oncoproteins enhance antigen presentation pathways, prominently through upregulated p16INK4a expression, potentially sensitizing tumors to immune checkpoint inhibition despite lower TMB and MSI statuses (12, 18). In our reported case, the presence of strong PD-L1 expression and HPV-16 positivity provided a justified rationale for using tislelizumab, supporting its efficacy in immunotherapy-resistant PSCC. Thus, despite relatively low TMB and MSI, immune checkpoint inhibition remains a valuable therapeutic strategy for HPV-positive PSCC, driven by viral oncogene-associated immunogenic alterations.

Timely psychological support helps patients resume normal daily routines. This case illustrates that optimal management of advanced penile cancer requires combining surgery with immunotherapy while also attending to the patient’s psychological welfare. A key limitation of this report is the restricted availability of pre-operative MRI, which hindered a comprehensive anatomical assessment of local tumour extent.

The case underscores the criticality of prompt and thorough diagnostic evaluation and therapeutic intervention for individuals with advanced penile cancer. Extensive lymph node dissection, coupled with judicious immunotherapeutic approaches, substantially ameliorates patient quality of life and augments survival prospects. In instances where patients exhibit intolerance to chemotherapy, PD-1 inhibitor immunotherapy emerges as a viable and efficacious alternative treatment modality. A multidisciplinary approach to treatment is necessary.

Despite these encouraging findings, there remain important considerations. First, large-scale prospective trials in PSCC are scarce, and the optimal sequencing or combination of immunotherapeutic agents with surgery, chemotherapy, or radiotherapy is yet to be conclusively determined. Second, while immune checkpoint inhibitors often exhibit a more tolerable toxicity profile compared to classic chemotherapy, immune-related adverse events can still pose significant clinical challenges. Third, robust biomarkers predicting treatment response—beyond PD-L1 expression and HPV status—are not fully established for PSCC and warrant further exploration to guide personalized therapeutic strategies.

## Conclusion

This case illustrates the potential utility of PD-1 inhibitors for the management of advanced HPV-16-related PSCC in patients who are suboptimal candidates for conventional chemotherapy. By integrating surgical resection with immunotherapy and comprehensive supportive care, clinicians may achieve meaningful disease control and maintain patient quality of life. Nevertheless, further multicenter studies and prospective trials are urgently needed to refine immunotherapeutic indications, elucidate predictive biomarkers, and establish standardized treatment algorithms for this rare yet clinically significant malignancy.

## Data Availability

The raw data supporting the conclusions of this article will be made available by the authors, without undue reservation.

## References

[1] Bleeker MCG Heideman DAM Snijders PJF Horenblas S Dillner J Meijer CJLM . Penile cancer: epidemiology, pathogenesis and prevention. World J Urol. (2009) 27:141–50. doi: 10.1007/s00345-008-0302-z 18607597

[2] Iorga L Marcu RD Diaconu C Stanescu AMA Stoian AP Mischianu DLD . Penile carcinoma and HPV infection. Exp Ther Med. (2020) 20:91–6. doi: 10.3892/etm.2019.8181 PMC7273896 32518604

[3] Vieira CB Feitoza L Pinho J Teixeira-Junior A Lages J Calixto J . Profile of patients with penile cancer in the region with the highest worldwide incidence. Sci Rep. (2020) 10:2965. doi: 10.1038/s41598-020-59831-5 32076037 PMC7031540

[4] Pagliaro LC Williams DL Daliani D Williams MB Osai W Kincaid M . Neoadjuvant paclitaxel, ifosfamide, and cisplatin chemotherapy for metastatic penile cancer: a phase II study. J Clin Oncol. (2010) 28:3851–7. doi: 10.1200/JCO.2010.29.5477 PMC2940402 20625118

[5] Udager AM Liu TY Skala SL Magers MJ McDaniel AS Spratt DE . Frequent PD-L1 expression in primary and metastatic penile squamous cell carcinoma: potential opportunities for immunotherapeutic approaches. Ann Oncol. (2016) 27:1706–12. doi: 10.1093/annonc/mdw216 PMC4999561 27217541

[6] Li N Xu T Zhou Z Li P Jia G Li X . Immunotherapy combined with chemotherapy for postoperative recurrent penile squamous cell carcinoma: a case report and literature review. Front Oncol. (2022) 12:837547. doi: 10.3389/fonc.2022.837547 35402270 PMC8984464

[7] Crispen PL Mydlo JH . Penile intraepithelial neoplasia and other premalignant lesions of the penis. Urol Clin North Am. (2010) 37:335–42. doi: 10.1016/j.ucl.2010.04.003 20674690

[8] Arya M Kalsi J Kelly J Muneer A . Malignant and premalignant lesions of the penis. BMJ. (2013) 346:f1149. doi: 10.1136/bmj.f1149 23468288

[9] Parza K Mustasam A Ionescu F Paravathaneni M Sandstrom R Safa H . The prognostic role of human papillomavirus and p16 status in penile squamous cell carcinoma: a systematic review. Cancers (Basel). (2023) 15:3713. doi: 10.3390/cancers15143713 37509374 PMC10378259

[10] Joshi SS Handorf E Strauss D Correa AF Kutikov A Chen DYT . Treatment trends and outcomes for patients with lymph node-positive cancer of the penis. JAMA Oncol. (2018) 4:643–9. doi: 10.1001/jamaoncol.2017.5608 PMC5885184 29494739

[11] Ansary TM Hossain MDR Komine M Ohtsuki M . Immunotherapy for the treatment of squamous cell carcinoma: potential benefits and challenges. Int J Mol Sci. (2022) 23:8530. doi: 10.3390/ijms23158530 35955666 PMC9368833

[12] Tang Y Hu X Wu K Li X . Immune landscape and immunotherapy for penile cancer. Front Immunol. (2022) 13:1055235. doi: 10.3389/fimmu.2022.1055235 36524123 PMC9745054

[13] Casagrande S Boscato GB Bertalot G Bortolotti R Racanelli V Caffo O . Immune-related adverse events due to cancer immunotherapy: immune mechanisms and clinical manifestations. Cancers (Basel). (2024) 16:1440. doi: 10.3390/cancers16071440 38611115 PMC11011060

[14] Nazha B Zhuang T Wu S Brown JT Magee D Carthon BC . Comprehensive genomic profiling of penile squamous cell carcinoma and the impact of human papillomavirus status on immune-checkpoint inhibitor-related biomarkers. Cancer. (2023) 129:3884–93. doi: 10.1002/cncr.34982 37565840

[15] Hrudka J Hojný J Prouzová Z Kendall-Bartu M Čapka D Zavillová N . High tumour mutational burden is associated with strong PD-L1 expression, HPV negativity, and worse survival in penile squamous cell carcinoma: an analysis of 165 cases. Pathology. (2024) 56:357–66. doi: 10.1016/j.pathol.2023.10.010 38161143

[16] Ahmed ME Falasiri S Hajiran A Chahoud J Spiess PE . The immune microenvironment in penile cancer and rationale for immunotherapy. J Clin Med. (2020) 9:3334. doi: 10.3390/jcm9103334 33080912 PMC7603091

[17] Kidd LC Chaing S Chipollini J Giuliano AR Spiess PE Sharma P . Relationship between human papillomavirus and penile cancer—implications for prevention and treatment. Transl Androl Urol. (2017) 6:791–802. doi: 10.21037/tau.2017.06.27 29184775 PMC5673821

[18] Long XY Zhang S Tang LS Li X Liu JY . Conversion therapy for advanced penile cancer with tislelizumab combined with chemotherapy: a case report and review of literature. World J Clin cases. (2022) 10:12305–12. doi: 10.12998/wjcc.v10.i33.12305 PMC9724507 36483823

